# *ZMYND11* p.Arg600Trp variant associated with a distinctive neurodevelopmental phenotype

**DOI:** 10.1038/s41439-026-00339-1

**Published:** 2026-03-12

**Authors:** Hidetaka Yoshimatsu, Jun Kido, Takaaki Sawada, Keishin Sugawara, Yohei Misumi, Yukina Hayashi, Atsushi Fujita, Naomichi Matsumoto, Mitsuharu Ueda, Kimitoshi Nakamura

**Affiliations:** 1 Division of Neonatology, Perinatal Center, Kumamoto City Hospital, Kumamoto, Japan; 2 Department of Pediatrics, Faculty of Life Sciences, Kumamoto University, Kumamoto, Japan; 3 Department of Pediatrics, Kumamoto University Hospital, Kumamoto, Japan; 4 Center for Clinical Genetics, Kumamoto University Hospital, Kumamoto, Japan; 5 Department of Neurology, Graduate School of Medical Sciences, Kumamoto University, Kumamoto, Japan; 6 Department of Human Genetics, Yokohama City University Graduate School of Medicine, Yokohama, Japan; 7 Department of Rare Disease Genomics, Yokohama City University Hospital, Yokohama, Japan; 8 Department of Clinical Genetics, Yokohama City University Hospital, Yokohama, Japan; 9 Medical Genome Center, National Center of Neurology and Psychiatry, Kodaira, Japan

**Keywords:** Genetic predisposition to disease, Medical genomics

## Abstract

Zinc finger MYND-type containing 11 (*ZMYND11*)-related neurodevelopmental disorder is an autosomal dominant condition caused by pathogenic variants in *ZMYND11*. Most previously reported patients harbor loss-of-function (LoF) variants, whereas missense variants are rare and their clinical and mechanistic characteristics remain insufficiently defined. Here we report a patient with a heterozygous *ZMYND11* c.1798C>T, p.(Arg600Trp) variant identified through the Initiative on Rare and Undiagnosed Diseases program. Detailed clinical evaluation, developmental assessment and whole-exome sequencing were performed. In addition, a systematic review of previously published *ZMYND11* cases was conducted to compare genotype–phenotype correlations between missense and LoF variants. The present patient showed global developmental delay, hypotonia, distinctive craniofacial features, microcephaly, short stature, cryptorchidism and right-sided inguinal hernia. Comparison with two previously reported individuals carrying the same c.1798C>T variant demonstrated consistent shared features, including microcephaly, broad nasal alae, short stature, cryptorchidism and nipple anomalies, findings that are not typically emphasized in LoF-associated cases. Aggregate analysis of reported 13 missense variants suggested higher frequencies of strabismus, hypotonia and severe intellectual disability compared with LoF variants, supporting the hypothesis that missense variants including c.1798C>T may define a partially distinct clinical subgroup. These findings expand the phenotypic spectrum associated with *ZMYND11* missense variants and suggest variant-specific clinical patterns, particularly for c.1798C>T, which may reflect a mechanism different from simple haploinsufficiency.

## Distinct features of ZMYND11 missense variants revealed

Zinc finger MYND-type containing 11 (*ZMYND11*) is a gene linked to a rare neurodevelopmental disorder. This disorder often causes developmental delays, intellectual disabilities and unique facial features. Most cases are due to loss-of-function variants, but missense variants are less understood. This study focuses on a specific missense variant in *ZMYND11*. The researchers studied a young boy carrying this variant, who exhibited symptoms such as developmental delay and distinctive facial features. They used genetic testing to identify the variant and compared his symptoms with other known cases. The study involved detailed clinical evaluations and genetic analysis using exome sequencing. The findings suggest that missense variants might lead to different symptoms than loss-of-function variants, such as more severe intellectual disabilities. The study highlights the need for more research to understand these differences better.

This summary was initially drafted using artificial intelligence, then revised and fact-checked by the author.

## Introduction

Zinc finger MYND-type containing 11 (*ZMYND11*)-related neurodevelopmental disorder is an autosomal dominant condition caused by pathogenic variants in the *ZMYND11* gene. This disorder is characterized primarily by developmental delay, intellectual disability, language impairment, autism spectrum disorder-like features, hypotonia and distinctive craniofacial morphology. Most reported cases involve loss-of-function (LoF) variants, such as copy number deletions or nonsense mutations^1,2^. By contrast, missense variants are rare, and their associated phenotypic spectrum and pathogenic mechanisms remain poorly understood.

Accumulating evidence suggests that a subset of *ZMYND11* missense variants may manifest clinical features that differ from those associated with haploinsufficiency variants such as frameshift or nonsense mutations^3^. The c.1798C>T p.(Arg600Trp) variant presented herein is identical to one such previously reported missense variant^4,5^, and our patient displayed several phenotypic findings that partially diverge from the classical features of *ZMYND11*-related disorders.

In this Article, we describe a patient carrying the *ZMYND11* (NM_001370100.5): c.1798C>T p.(Arg600Trp) variant identified through the Initiative on Rare and Undiagnosed Diseases (IRUD)^6,7^. The aims of this study are: (1) to characterize the phenotype associated with this missense variant in comparison with the previously reported case harboring the same variant, as well as with other published cases of *ZMYND11*-related neurodevelopmental disorder; and (2) to systematically compare reported missense and LoF variants in *ZMYND11* to evaluate potential relationships between genotype and phenotype.

## Materials and methods

### Patient recruitment and clinical evaluation

This patient was enrolled in the IRUD in Japan. A comprehensive clinical evaluation was performed according to the standardized IRUD protocol by the attending physicians in collaboration with relevant specialists. Retrospective data collection included birth and perinatal history, physical and neurological examinations, developmental history, behavioral features, neuroimaging findings, laboratory tests and chromosomal analyses. Developmental assessments were performed using age-appropriate standardized instruments.

### Genetic analysis

Genetic analysis was performed according to the IRUD standard protocol. Exome sequencing was conducted as described previously^8,9^. The mean depth of coverage for RefSeq coding sequences was 60.88×, with 97.0% of all coding regions covered by at least 10 reads. The entire coding region of *ZMYND11* was covered by at least 20 reads. Variant nomenclature followed the guidelines established by the Human Genome Variation Society (http://varnomen.hgvs.org/)^10^, and variants were described at the protein level. The reference sequence used for annotation was NM_001370100.5. The clinical significance of each variant was assessed with reference to the ClinVar database (http://www.ncbi.nlm.nih.gov/clinvar)^11^, and variant interpretation followed the American College of Medical Genetics and Genomics/Association for Molecular Pathology guidelines. For missense variants, the potential impact of amino acid substitutions on *ZMYND11* function was predicted using the bioinformatic tool PolyPhen-2 (http://genetics.bwh.harvard.edu/pph2)^12^. The candidate *ZMYND11* variant and the familial segregation were validated by trio-based Sanger sequencing. Polymerase chain reaction (PCR) was performed using TaKaRa Ex Taq HS (Takara Bio), and sequencing was conducted on a SeqStudio Genetic Analyzer (Thermo Fisher Scientific). Primers were designed using Primer3Plus (https://www.primer3plus.com/) with standard parameters, and specificity was confirmed using UCSC In-silico PCR (https://genome-asia.ucsc.edu/cgi-bin/hgPcr). The final primer sequences were forward: 5′- ACTGCCCCGATTTCTTACCT-3′ and reverse: 5′- TGAAAAACTTCGGAGGAGCA-3′.

### Systematic literature review

An electronic literature search of the PubMed database was performed for current and past findings of the *ZMYND11* mutation using the keywords *‘ZMYND11’*, ‘BS69’, ‘mutation’, ‘variant’, ‘10p15.3 deletion syndrome’, ‘clinical phenotype’ and so on. When there were overlaps between multiple studies, the more relevant articles were used. We evaluated the genotype and clinical phenotype of patients with the *ZMYND11* variant reported in nine papers^1,3–5,13–17^.

### Data extraction and phenotypic comparison

For each reported case, we extracted clinical information including age, sex, variant details (nucleotide change, amino acid change, variant position and variant type), developmental and motor outcomes, behavioral features, dysmorphic findings, epilepsy, microcephaly, small for gestational age (SGA), short stature, strabismus and cryptorchidism. The phenotypic features associated with the missense variant identified in our patient (p.Arg600Trp) were compared with those of previously reported cases. Furthermore, we examined phenotypic differences between missense variants and LoF variants.

## Results

### Case report

**Fig. 1:**
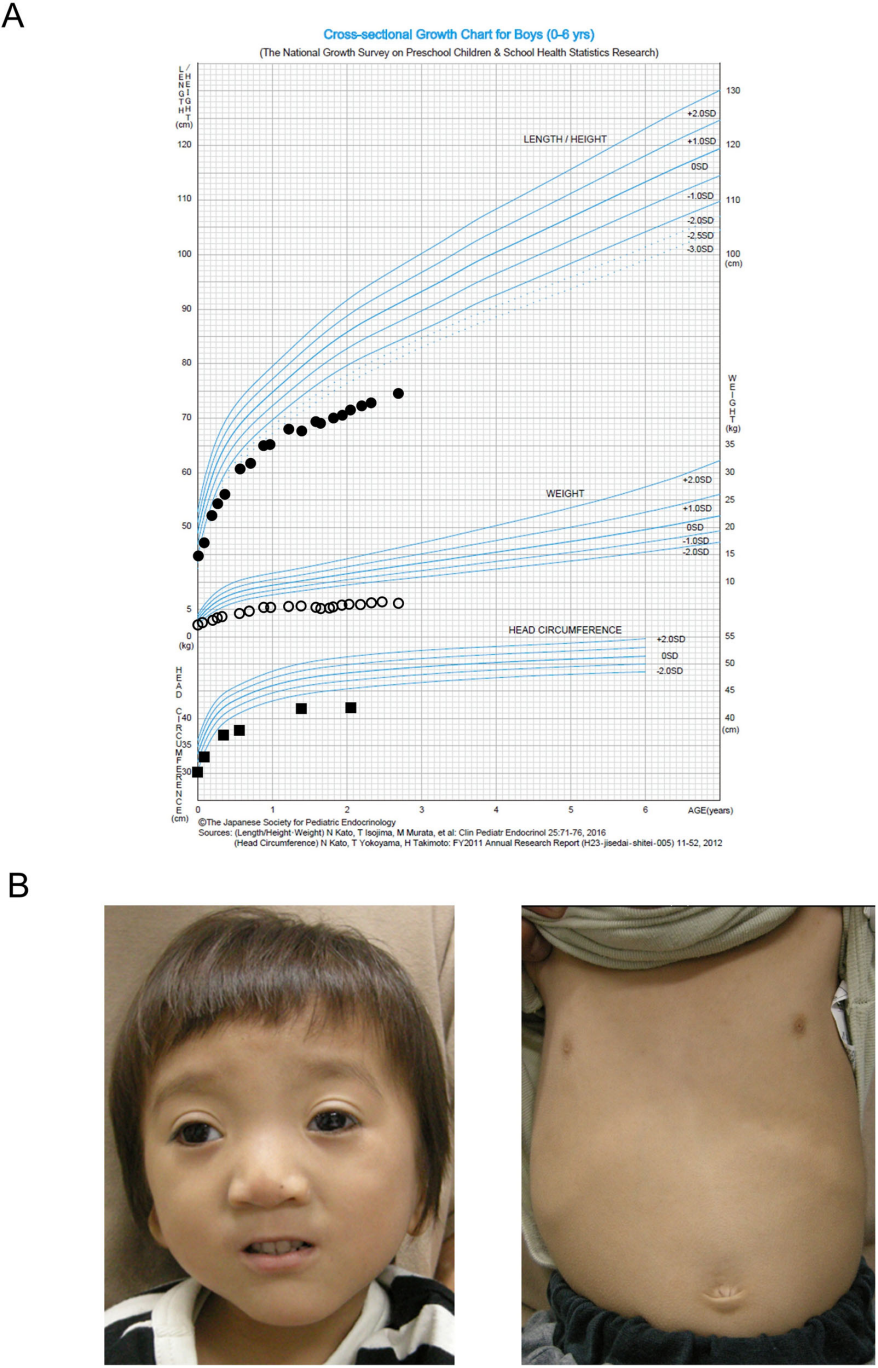
Clinical manifestations of the patient. **A** Growth curve of the patient. **B** Craniofacial and thoracic features of the patient.

This patient is a male infant born at 39 weeks and 1 day of gestation via vaginal delivery. Birth weight was 2220 g (−2.64 standard deviation (s.d.)), length 44.5 cm (−2.2 s.d.) and head circumference 30 cm (−2.4 s.d.), consistent with SGA (Fig. 1A). Apgar scores were 8 and 9 at 1 and 5 min, respectively. From birth, he exhibited hypotonia, respiratory distress and poor feeding, and was transferred to our Neonatal Intensive Care Unit on day 2 of life. The prenatal course was notable for fetal growth restriction, with no reported maternal complications. Family history revealed a sibling with poor feeding and speech articulation difficulties who had received developmental support, although no motor or cognitive impairments were reported.

Physical examination during the neonatal period revealed multiple craniofacial anomalies, including hypopigmented hair, low-set ears, hypertelorism, a broad nasal bridge, small nares with broad alae nasi, a small mouth, mandibular prognathism and a narrow mandible; right-sided cryptorchidism was also noted. Respiratory status stabilized under high-flow nasal cannula therapy, with successful weaning achieved by day 22. Full oral feeding was established on day 19, and he was discharged on day 35 with a body weight of 2700 g.

Brain magnetic resonance imaging performed at 6 months showed no overt structural abnormalities. He had achieved head control at the age of 14 months and rolling over at 1 year 4 months; however, independent sitting had not yet been achieved. At the age of 2 years, developmental testing using the Kyoto Scale of Psychological Development showed a total developmental quotient of 25 (postural-motor 24, cognitive-adaptive 24, language-social 40), consistent with moderate-to-severe global developmental delay.

Additional comorbidities included right inguinal hernia, exotropia and hyperopic astigmatism, for which corrective glasses were prescribed. Behaviorally, he exhibited self-injurious behaviors, such as striking his face when distressed, probably related to absent expressive language.

**Fig. 2:**
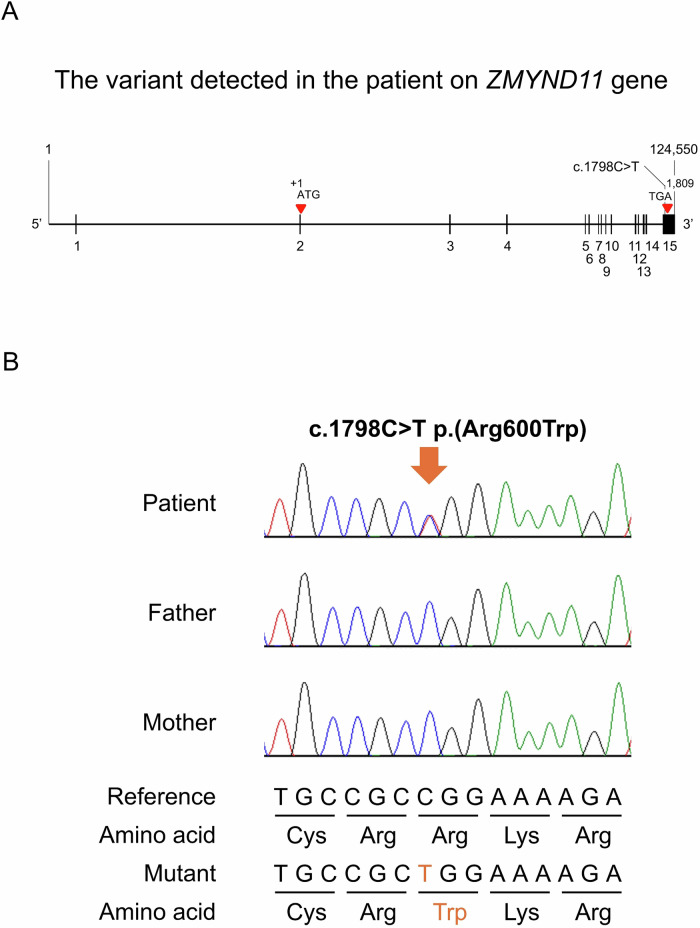
Variant of c.1798C>T p.(Arg600Trp) in *ZMYND11.* **A** Schematic representation of the *ZMYND11* gene and the location of the identified variant. The missense variant p.(Arg600Trp) is located in the exon 15 of *ZMYND11*. **B** Sanger sequencing in this family.

Genetic evaluations included karyotyping (G-banding) on day 11, which revealed a normal 46, XY complement; fluorescence in situ hybridization analysis of chromosome 4 at 11 months was negative; and chromosomal microarray at 13 months showed no abnormalities. The genetic analysis through the IRUD identified a de novo heterozygous missense variant of *ZMYND11* (NM_001370100.5: c.1798C>T p.(Arg600Trp)) (Fig. 2). At present, at 2 years and 9 months of age, he can maintain a sitting position but cannot stand or walk independently (Fig. 1A, B).

### Systematic literature review

Genotypic and phenotypic data of patients with *ZMYND11* variants, including the present case, were compiled from 50 reported cases (Table 1 and Supplementary Data 1). Among these patients, missense variants were identified in 13 cases (26%), frameshift variants in 17 cases (34%), in-frame variants in 2 cases (4%), nonsense variants in 15 cases (30%) and splice-site variants in 3 cases (6%).

**Table 1:** Summary of reported *ZMYND11* variants and associated clinical features.

Feature	p.Arg600Trp (*n* = 3)	Other *ZMYND*11 missense variants (*n* = 10)	LoF (*n* = 37)	Total
ID/GDD	3/3 (100%)	7/8 (88%)	32/32 (100%)	42/43 (98%)
Severe ID	2/2 (100%)	1/2 (50%)	2/17 (12%)	5/21 (24%)
Moderate ID	0/2	1/2 (50%)	2/17 (12%)	3/21 (14%)
Mild ID	0/2	0/2	13/17(77%)	13/21 (62%)
GMD	3/3 (100%)	5/7 (71%)	17/20 (85%)	25/30 (83%)
SD	2/2 (100%)	8/8 (100%)	29/29 (100%)	39/39 (100%)
BD	1/2 (50%)	3/5 (60%)	24/26 (92%)	28/33 (85%)
AD/hyperactivity/impulsivity	1/2 (50%)	4/6 (67%)	11/19 (58%)	16/27 (59%)
Aggression/anger	1/2 (50%)	2/5 (40%)	11/19 (58%)	14/26 (54%)
Autism/AT	0/2	3/5 (60%)	11/28 (39%)	14/35 (40%)
FP	3/3 (100%)	9/10 (90%)	27/35 (77%)	39/48 (81%)
Hypotonia	2/2 (100%)	7/8 (88%)	10/21 (48%)	19/31 (61%)
Epilepsy	1/3 (33%)	7/10 (70%)	14/34 (41%)	22/47 (47%)
Microcephaly	3/3 (100%)	2/10 (20%)	4/37 (11%)	9/50 (18%)
SGA	2/2 (100%)	0/5	3/12 (25%)	5/19 (26%)
SS	2/2 (100%)	2/5 (40%)	4/15 (27%)	8/22 (36%)
Strabismus	1/2 (50%)	5/6 (83%)	2/15 (13%)	8/23 (35%)
Cryptorchidism	2/2 (100%)	1/5 (20%)	0/12	3/19 (16%)
Nipple anomalies	2/2 (100%)	NA	NA	2/2 (100%)

Denominators indicate the number of individuals for whom the corresponding clinical information was available. *AD* attention deficits, *AT* autistic trait, *BD* behavioral difficulties, *FP* feeding problem, *GDD* global developmental delay, *GMD* gross motor delay, *ID* intellectual disability, *m* months, *NR* not reported, *SD* speech delay, *SS* short stature.

Developmental delay (28/28) and speech delay (39/39) were presented in all evaluable cases. Intellectual disability was observed in nearly all patients (34/35). Other frequently reported clinical features included dysmorphic craniofacial characteristics (39/48), motor delay (25/30) and behavioral difficulties (28/33). Additional findings included attention-deficit/hyperactivity/impulsivity (16/27), aggression or anger (14/26), autism spectrum features (14/35), epilepsy (22/47), hypotonia (19/31) and feeding difficulties (15/22), each occurring in approximately half of these patients.

### p.Arg600Trp variant in the present case

Comparison with the two previously reported cases carrying the same variant revealed several shared features: characteristic craniofacial dysmorphism (3/3), feeding difficulties (2/3), developmental delay (2/2), gross motor delay (3/3), speech delay (2/2), intellectual disability (2/2) and hypotonia (2/2) (Table 1 and Supplementary Data 1). These findings are consistent with the commonly reported features of *ZMYND11*-related neurodevelopmental disorder.

By contrast, several additional features not consistently observed in *ZMYND11*-related disorders were present in all three cases, including microcephaly (3/3), SGA (2/2), short stature (2/2), cryptorchidism (2/2) and distinct morphological features such as broad nasal alae, inverted nipples and widely spaced nipples.

### Analysis of missense variants

Among patients with missense variants, specific trends were noted: strabismus was observed in 6 of 8 cases (compared with 2/15 in LoF variants), hypotonia in 9 of 10 cases (versus 10/21 in LoF variants) and a tendency toward more severe intellectual disability than that seen in patients with LoF variants (Table 1 and Supplementary Data 1).

## Discussion

We described a patient with *ZMYND11*-related neurodevelopmental disorder, presenting with developmental delay, hypotonia, distinctive craniofacial features, microcephaly and short stature, as well as inguinal hernia and cryptorchidism.

*ZMYND11* functions as a tumor suppressor and modulates transcription by binding H3K36me3 and regulating RNA polymerase II-mediated transcriptional elongation^18^. Research using murine models has demonstrated that loss of *ZMYND11* disrupts neurogenesis and neuronal development during embryogenesis, impairing neuronal maturation^19^. These findings underscore its pivotal role in neurodevelopment and suggest that perturbations in *ZMYND11* may contribute to neurodevelopmental disorders, including intellectual disability.

*ZMYND11* possesses a multidomain architecture consisting of Bromo–ZnF–PWWP–MYND domains. The PWWP domain binds to H3K36me3-enriched chromatin, while the MYND domain recruits transcriptional regulators, including EZH2, HDAC1, Brg1 and E2F6, to target genes, thereby mediating transcriptional repression^20^. The MYND domain contains a zinc-binding motif essential for interactions with partner proteins, and Arg600 has been identified as a key residue for ligand binding^21^. Therefore, the p.Arg600Trp variant in the present case may impair *ZMYND11*-mediated transcriptional repression by interfering with the proper formation of the repressive complex, which is consistent with the proposed pathophysiology of *ZMYND11*-related neurodevelopmental disorders.

The c.1798C>T p.(Arg600Trp) is registered in ClinVar as ‘pathogenic/likely pathogenic’ and not registered in the gnomAD^22^ or the Tohoku Medical Megabank^23^ genome databases. In silico analysis using PolyPhen-2^12^ predicted it as ‘probably damaging’ (score 1.000). According to the American College of Medical Genetics and Genomics/Association for Molecular Pathology guidelines, this variant is classified as ‘pathogenic’ based on the criteria PS1, PM2, PS2 and PP3^24^. These findings consistently suggest that this variant is pathogenic.

Most previously reported cases of *ZMYND11*-related neurodevelopmental disorders are caused by LoF variants, with haploinsufficiency considered the primary pathogenic mechanism^1,2^. By contrast, missense variants, such as that observed in the present case, have been reported less frequently. Yates et al. suggested that certain missense variants may result in phenotypes distinct from those caused by LoF variants^3^, whereas a cohort study of 47 cases by Oates et al. did not reveal a clear correlation^15^.

The present patient carries the *ZMYND11* c.1798C>T p.(Arg600Trp) variant. Shared features with two previously reported cases include cryptorchidism, microcephaly, broad nasal alae, short stature and nipple anomalies. These features have been reported in only a minority of previously described *ZMYND11*-related neurodevelopmental disorders and may be more frequently observed in individuals carrying the p.(Arg600Trp) variant.

The patient also presented with right-sided inguinal hernia. *ZMYND11* is expressed in multiple tissues, including the nervous, immune and genitourinary systems^13^, suggesting that this feature may represent an expansion of the disorder’s phenotypic spectrum.

Comparison of variant types showed that missense mutations are more frequently associated with severe intellectual disability, hypotonia and strabismus than LoF variants. This suggests that missense mutations may form a distinct clinical subgroup within *ZMYND11*-related disorders, potentially involving alternative pathogenic mechanisms, including gain-of-function effects.

The available data raise the possibility that the c.1798C>T p.(Arg600Trp) variant is associated with clinical features that differ, at least in part, from the established *ZMYND11*-related phenotype. However, this report is based on a single case, and only two additional cases with the same variant have been described, limiting the certainty of a variant-specific phenotype. In addition, the previously reported cases vary in the level of clinical detail provided, and functional analyses have not been performed. Further accumulation of cases and functional studies are needed to clarify the pathogenic mechanisms and clinical significance of this variant.

## Conclusion

This report provides a detailed description of the clinical features of a patient carrying the *ZMYND11* c.1798C>T (p.Arg600Trp) variant. This variant may be associated with clinical features that are somewhat distinct from those typically observed in *ZMYND11*-related disorders. Missense variants tend to be associated with higher frequencies of strabismus, hypotonia and severe intellectual disability compared with LoF variants. Further accumulation of cases and functional analyses are expected to clarify the pathogenic mechanisms and clinical significance of this variant.

## Supplementary information

Supplementary Data 1
