# Supplementary material for: ZMYND11 p.Arg600Trp variant associated with a distinctive neurodevelopmental phenotype
Source: Hum Genome Var. 2026 Mar 12;13:7. doi: 10.1038/s41439-026-00339-1 (PMC13000240; doi:10.1038/s41439-026-00339-1)
Supplement: Supplementary file 1 — Supplementary Data 1 [file 41439_2026_339_MOESM1_ESM.docx]

Supplementary Data 1. Summary of reported *ZMYND11* variants and associated clinical features

| ***Number*** | ***Nucleic acid*** | ***Ami- acid*** | ***Location*** | ***Variant type*** | ***Age reported*** | ***Gender*** | ***FP*** | ***Dysmorphic*** | ***GDD*** | ***ID*** | ***GMD*** | ***SD*** | ***BD*** | ***AD/hyperactivity /impulsivity*** | ***Aggression /anger*** | ***Autism/AT*** | ***Hypotonia*** | ***Epilepsy*** | ***HC*** | ***SGA*** | ***SS*** | ***Strabismus*** | ***Cryptorchidism*** | ***Nipple anomalies*** | ***Reference*** |
| --- | --- | --- | --- | --- | --- | --- | --- | --- | --- | --- | --- | --- | --- | --- | --- | --- | --- | --- | --- | --- | --- | --- | --- | --- | --- |
| **1** | **c.1798C>T**  **(This case)** | **p.Arg600Trp** | **Exon 15** | **Missense** | **2y11m** | **M** | **+** | **+** | **+** | **NR** | **+** | **+** | **+** | **+** | **+** | **-** | **+** | **-** | **MiC** | **+** | **+** | **+** | **+** | **+** |  |
| **2** | **c.1798C>T** | **p.Arg600Trp** | **Exon 15** | **Missense** | **7y** | **M** | **+** | **+** | **+** | **Severe** | **+** | **+** | **NR** | **NR** | **NR** | **NR** | **+** | **-** | **MiC** | **+** | **+** | **-** | **+** | **+** | **4** |
| **3** | **c.1798C>T** | **p.Arg600Trp** | **Exon 15** | **Missense** | **34y** | **F** | **NR** | **+** | **NR** | **Severe** | **+** | **NR** | **-** | **-** | **-** | **-** | **NR** | **+** | **MiC** | **NR** | **NR** | **NR** | **-** | **NR** | **5** |
| 4 | c.76C>T | p.Arg26Trp | Exon 2 | Missense | 8y | M | NR | **+** | NR | **+** | NR | NR | NR | NR | NR | NR | **+** | - | MaC | NR | NR | NR | **+** | NR | 12 |
| 5 | c.926G>A | p.Arg309His | Exon 10 | Missense | 5y | F | NR | **+** | NR | NR | **+** | **+** | - | - | - | - | **+** | **+** | NoC | NR | NR | **+** | - | NR | 12 |
| 6 | c.1246G>A | p.Glu416Lys | Exon 13 | Missense | 2y5m | M | **+** | **+** | **+** | NR | **+** | **+** | **+** | - | - | NR | **+** | - | MaC | - | - | **+** | NR | NR | 3 |
| 7 | c.1253T>G | p.Val418Gly | Exon 13 | Missense | 2y5m | F | **+** | **+** | **+** | NR | **+** | **+** | NR | NR | NR | NR | **+** | + | MiC | - | - | **+** | - | NR | 11 |
| 8 | c.1262G>A | p.Ser421Asn | Exon 13 | Missense | 5y6m | M | **+** | **+** | **+** | NR | **+** | **+** | + | **+** | NR | NR | **+** | - | NoC | - | **+** | **+** | NR | NR | 11 |
| 9 | c.1262G>A | p.Ser421Asn | Exon 13 | Missense | 24y | F | **+** | **+** | **+** | Severe | **+** | **+** | - | **+** | - | - | **+** | **+** | MiC | - | NR | - | - | NR | 10 |
| 10 | c.1685G>C | p.Trp562Ser | Exon 14 | Missense | 7y | M | NR | **+** | NR | - | - | **+** | NR | **+** | NR | NR | NR | **+** | NoC | NR | NR | NR | NR | NR | 12 |
| 11 | c.1720T>C | p.Cys574Arg | Exon 15 | Missense | 15y | M | - | **+** | **+** | Moderate | - | **+** | **+** | **+** | **+** | **+** | - | **+** | MaC | - | - | **+** | NR | NR | 3 |
| 12 | c.1724G>A | p.Cys575Tyr | Exon 15 | Missense | NR | NR | NR | - | NR | NR | NR | NR | NR | NR | + | + | + | **+** | NR | NR | NR | NR | NR | NR | 12 |
| 13 | c.1793G>C | p.Cys598Ser | Exon 15 | Missense | 10y | F | NR | **+** | **+** | NR | NR | + | NR | NR | NR | + | NR | **+** | MaC | NR | + | NR | - | NR | 12 |
| 14 | c.117-2A>T | unknown | Intron 2 | Splice acceptor | 8y | F | - | - | NR | Mild | - | NR | **+** | **+** | - | - | - | **+** | NoC | NR | NR | - | - | NR | 3 |
| 15 | c.1159-2A>G | unknown | Intron 11 | Splice acceptor | NR | NR | NR | **+** | **+** | **+** | NR | **+** | NR | NR | NR | **+** | NR | - | NR | NR | NR | NR | NR | NR | 12 |
| 16 | c.1687-1G>A | unknown | Intron 14 | Splice acceptor | NR | NR | NR | **+** | NR | NR | **+** | **+** | NR | NR | NR | NR | NR | NR | NR | NR | NR | NR | NR | NR | 12 |
| 17 | c.206dupT | p.Thr70Asnfs*12 | Exon 3 | Frame shift | 17y | F | NR | **+** | NR | Mild | **+** | **+** | **+** | - | - | **+** | **+** | - | NoC | - | - | - | - | NR | 1 |
| 18 | c.383del | p.Ser128Leufs*42 | Exon 4 | Frame shift | NR | NR | **+** | **+** | **+** | Severe | NR | NR | NR | NR | **+** | NR | **+** | **+** | MaC | NR | NR | NR | NR | NR | 13 |
| 19 | c.454_455insC | p.Asn152Thrfs*26 | Exon 5 | Frame shift | 22y | M | NR | - | NR | **+** | **+** | **+** | **+** | NR | NR | NR | - | **+** | NoC | NR | NR | **+** | NR | NR | 1 |
| 20 | c.561del | p.Met187Ilefs*19 | Exon 6 | Frame shift | 25y | M | NR | **+** | NR | Mild | **+** | **+** | **+** | **+** | **+** | - | NR | - | NoC | **+** | - | - | NR | NR | 1 |
| 21 | c.705_708del | p.Glu236Lysfs*52 | Exon 8 | Frame shift | 8y | M | **+** | - | + | Mild | + | + | **+** | **+** | - | - | **+** | - | NoC | **+** | - | - | NR | NR | 3 |
| 22 | c.737_738del | p.Lys246Argfs*5 | Exon 8 | Frame shift | 4y5m | M | NR | **+** | **+** | Moderate | NR | **+** | NR | NR | **+** | **+** | NR | **+** | NoC | NR | NR | NR | NR | NR | 12 |
| 23 | c.737_738del | p.Lys246Argfs*5 | Exon 8 | Frame shift | 13y1m | M | NR | - | NR | Mild | NR | NR | NR | NR | NR | **+** | NR | **+** | NR | NR | NR | NR | NR | NR | 12 |
| 24 | c.1129del | p.Ser377Profs*11 | Exon 11 | Frame shift | 1y7m | M | NR | NR | NR | Mild | NR | NR | NR | NR | NR | NR | NR | **+** | NR | NR | NR | NR | NR | NR | 12 |
| 25 | c.1129del | p.Ser377Profs*11 | Exon 11 | Frame shift | 4y | M | - | - | **+** | Mild | **+** | **+** | - | - | - | - | - | - | MiC | - | **+** | NR | NR | NR | 3 |
| 26 | c.1246_1247del | p.Glu416Serfs*5 | Exon 13 | Frame shift | NR | M | NR | **+** | NR | Severe | NR | **+** | NR | NR | NR | **+** | **+** | **+** | MiC | - | **+** | NR | NR | NR | 1 |
| 27 | c.1317_1320del | p.Thr440Argfs*3 | Exon 13 | Frame shift | 13y8m | M | **+** | **+** | **+** | Moderate | **+** | **+** | **+** | **+** | **+** | - | - | - | NR | NR | NR | NR | NR | NR | 3 |
| 28 | c.1438del | p.Asp480Thrfs*3 | Exon 13 | Frame shift | NR | NR | NR | **+** | NR | **+** | NR | NR | **+** | NR | NR | NR | NR | - | MiC | NR | NR | NR | NR | NR | 14 |
| 29 | c.1525_1526del | p.Lys509Glufs*6 | Exon 14 | Frame shift | 18y | M | **+** | - | **+** | NR | - | **+** | **+** | **+** | - | - | **+** | - | NR | NR | NR | NR | NR | NR | 3 |
| 30 | c.1573dup | p.Asp525Glyfs*5 | Exon 14 | Frame shift | 2y7m | F | - | **+** | **+** | Moderate | **+** | **+** | **+** | - | - | - | - | - | MiC | **+** | **+** | - | - | NR | 3 |
| 31 | c.1581dup | p.Cys528Metfs*2 | Exon 14 | Frame shift | 4y1m | NR | NR | **+** | **+** | NR | **+** | **+** | **+** | NR | NR | - | NR | - | NR | NR | NR | NR | NR | NR | 12 |
| 32 | c.1581dup | p.Cys528Metfs*2 | Exon 14 | Frame shift | 9y5m | F | NR | **+** | **+** | Mild | **+** | **+** | **+** | + | NR | - | NR | - | NR | NR | NR | NR | - | NR | 12 |
| 33 | c.1581dup | p.Cys528Metfs*2 | Exon 14 | Frame shift | 25y | F | NR | **+** | **+** | Mild | NR | NR | **+** | NR | NR | - | NR | - | NR | NR | NR | NR | - | NR | 12 |
| 34 | c.1759_1761  del | p.Gln587del | Exon 15 | In-flame | NR | F | **+** | **+** | **+** | Mild | NR | **+** | **+** | **+** | NR | - | **+** | - | NoC | - | - | - | - | NR | 1 |
| 35 | c.1759_1761  del | p.Gln587del | Exon 15 | In-flame | NR | NR | **+** | **+** | NR | Mild | NR | **+** | **+** | NR | NR | NR | **+** | - | NR | NR | NR | NR | NR | NR | 12 |
| 36 | c.22C>T | p.Arg8* | Exon 2 | Nonsense | NR | NR | NR | **+** | NR | NR | NR | **+** | NR | NR | NR | NR | NR | **+** | MaC | NR | NR | NR | NR | NR | 12 |
| 37 | c.46C>T | p.Gln16* | Exon 2 | Nonsense | NR | NR | **+** | **+** | **+** | NR | NR | **+** | **+** | NR | NR | **+** | **+** | - | NR | NR | NR | NR | NR | NR | 12 |
| 38 | c.46C>T | p.Gln16* | Exon 2 | Nonsense | 3y | F | **+** | **+** | **+** | NR | **+** | **+** | **+** | **+** | - | - | **+** | - | NoC | - | - | - | - | NR | 3 |
| 39 | c.82C>T | p.Gln28* | Exon 2 | Nonsense | NR | NR | NR | **+** | NR | NR | NR | **+** | NR | NR | NR | **+** | NR | **+** | NR | NR | + | NR | NR | NR | 12 |
| 40 | c.630C>G | p.Tyr210* | Exon 7 | Nonsense | 5y10m | F | NR | **+** | **+** | Mild | **+** | **+** | **+** | - | **+** | **+** | - | **+** | NoC | - | - | - | - | NR | 3 |
| 41 | c.709C>T | p.Gln237* | Exon 8 | Nonsense | 10y | F | NR | - | NR | NR | NR | **+** | NR | **+** | NR | **+** | NR | **+** | NoC | NR | - | NR | - | NR | 12 |
| 42 | c.976C>T | p.Gln326* | Exon 11 | Nonsense | 41y | M | NR | **+** | NR | Mild | NR | **+** | **+** | NR | NR | NR | NR | NR | NoC | NR | - | NR | NR | NR | 1 |
| 43 | c.1072C>T | p.Arg358* | Exon 11 | Nonsense | NR | NR | NR | NR | NR | **+** | NR | NR | NR | NR | NR | NR | NR | NR | NR | NR | NR | NR | NR | NR | 12 |
| 44 | c.1089G>A | p.Trp363* | Exon 11 | Nonsense | 8y | M | NR | **+** | **+** | Mild | **+** | **+** | **+** | - | + | - | - | - | NoC | - | - | **+** | NR | NR | 3 |
| 45 | c.1531C>T | p.Gln511* | Exon 14 | Nonsense | 8y | M | **+** | **+** | **+** | Mild | **+** | **+** | **+** | - | **+** | **+** | **+** | - | MaC | - | NR | - | NR | NR | 3 |
| 46 | c.1666C>T | p.Gln556* | Exon 14 | Nonsense | 21y | M | NR | **+** | NR | Moderate | **+** | **+** | **+** | NR | **+** | **+** | NR | **+** | MaC | NR | NR | NR | NR | NR | 12 |
| 47 | c.1756C>T | p.Gln586* | Exon 15 | Nonsense | 22y | F | - | **+** | **+** | **+** | - | + | **+** | **+** | **+** | - | - | **+** | NoC | - | - | - | - | NR | 3 |
| 48 | c.1756C>T | p.Gln586* | Exon 15 | Nonsense | 47y | F | NR | - | NR | **+** | NR | NR | - | - | - | - | - | **+** | NoC | NR | - | - | - | NR | 3 |
| 49 | c.1756C>T | c.1756C>T | Exon 15 | Nonsense | 17y | M | - | **+** | **+** | **+** | **+** | **+** | **+** | **+** | **+** | - | - | - | NR | NR | NR | - | NR | NR | 3 |
| 50 | c.1756C>T | c.1756C>T | Exon 15 | Nonsense | 20y | F | - | **+** | **+** | **+** | **+** | **+** | **+** | - | **+** | - | - | - | NR | NR | NR | - | - | NR | 3 |

AD, attention deficits; AT, autistic trait; BD, behavioral difficulties; FP, feeding problem; GDD, global developmental delay; GMD, gross motor delay; HC, head　circumference; ID, intellectual disability; MaC, macrocephaly; MiC, microcephaly; m, months; NoC, normocephaly; NR, not reported; SD, speech delay; SGA, small for gestational age; SS, short stature; y, years.
